# *Streptococcus thermophilus* JM905—Strain Carbon Source Utilization and Its Fermented Milk Metabolic Profile at Different Fermentation Stages

**DOI:** 10.3390/foods12193690

**Published:** 2023-10-08

**Authors:** Yu Li, Ye Wang, Baolei Li, Baochao Hou, Weilian Hung, Jian He, Yujun Jiang, Yu Zhang, Chaoxin Man

**Affiliations:** 1 Key Laboratory of Dairy Science Ministry of Education, College of Food Science and Engineering, Northeast Agricultural University, Harbin 150030, China; hrbliyu1007@163.com (Y.L.); wyyyee2000@163.com (Y.W.); yujun_jiang@163.com (Y.J.); jessedevil@163.com (Y.Z.); 2 National Center of Technology Innovation for Dairy, Shanghai 201111, China; libaolei@yili.com (B.L.); houbaochao@yili.com (B.H.); hongweilian@yili.com (W.H.); hejian@yili.com (J.H.)

**Keywords:** *Streptococcus thermophilus*, fermented milk, fermentation characteristics, metabolism, quality

## Abstract

The metabolic utilization of different carbon sources by *Streptococcus thermophilus* JM905(*S. thermophilus* JM905) was determined using a high-throughput microbial phenotyping system, and changes in fermentation characteristics of *S. thermophilus* JM905 fermented milk were investigated at different fermentation periods, with changes in pH, water-holding capacity, viscosity, nuisance odor, and viable bacteria count being used to define the fermentation characteristics of the strain. Changes in the key metabolites, 2-hydroxybutyric acid, folic acid, L-lactic acid, D-glycerol-D-galactose-heptanol, (R)-leucine, L-aspartic acid, L-proline, D-arginine, L-isoleucine, hydra starch, L-lysine, L-tryptophan, and D-galactose, were clarified. Correspondingly, the fermented milk protein, amino acid, and fermented milk fat quality nutrient contents were determined to be 3.78 ± 0.054 g per 100 g, 3.405 ± 0.0234 g per 100 mL, and 0.161 ± 0.0030 g per 100 g, respectively. This study addressed strain carbon source utilization, changes in fermentation characteristics and metabolites during fermentation, with the aim of investigating the link between fermentation characteristics and metabolite quality components of *Streptococcus thermophilus* JM905 and its fermented milk with fermentation potential and to provide a useful reference for the screening of superior fermentation strains.

## 1. Introduction

Fermented milk is a product made from raw milk or milk powder that has been sterilized and fermented to reduce pH [1]. Lactic acid bacteria are mainly selected for fermentation and, to a lesser extent, yeasts and molds [2]. Compared to unfermented dairy products, they are characterized by the addition of a single probiotic or multiple probiotics for fermentation culture. The properties of different kinds of fermented milk are improved by adding appropriate amounts of ingredients, such as thickeners, functional substances, and natural plant ingredients, to improve the texture, consistency, dietary fiber content, quality features, and taste and flavor of different kinds of fermented milk. The effect of fermentation on the food matrix is manifested by the availability of a variety of nutrients and functions that enrich the fermented matrix and ultimately give the food a multi-layered taste and good organoleptic qualities in line with today’s demand for new foods [3]. Among fermented foods, fermented milk has attracted much attention due to its great digestibility, rich taste, and ability to alleviate lactose intolerance, and accordingly, multidimensional studies have been carried out on its technical and industrial uses. Currently, most of the commercially available fermented milk is produced on the basis of cow’s milk substrate, which is more acceptable to consumers when considering the nutritional value and benefits compared to other substrates due to its safety, high nutritional value [4], popularity in terms of taste and flavor, wide availability, as well as systematic development and promotion [5]. More and more consumers are recognizing the benefits of fermented products, and the production and consumption of fermented milk are growing dramatically worldwide [6].

Different kinds of fermented milk often have different fermentation characteristics, textures, and flavors, and the corresponding fermented products also show differences in composition, depending on a variety of factors in the fermentation preparation process, but the essence lies in the different fermentation characteristics of the lactic acid strains added and the differences in the final quality of the fermented milk. *S. thermophilus* is more suitable for growth in the milk environment due to its long period of genetic remodeling. This, in turn, has resulted in altered carbohydrate (carbon source) utilization by *S. thermophilus* in the milk environment. In particular, a strain dependence was shown for the main uptake and metabolic utilization of sugars (sucrose, glucose, galactose, fructose, etc.), with lactose being preferred. *S. thermophilus* utilizes and metabolizes carbon sources through a homo-fermentation pathway, followed by glycolysis, pyruvate metabolism, and other pathways, thus generating various metabolites to complete the fermentation process. Differences in the utilization of different carbon sources by *S. thermophilus* also lead to growth differences. During the fermentation process, fermented milk exhibits different fermentation characteristics at different fermentation stages, and the corresponding trends of fermentation characteristics are closely related to the final fermented products. In the fermentation system, the unique fermentation characteristics of different strains directly affect the content and metabolite composition of fermentation products. With the industrialization of fermented products and research efforts in recent years, compound strains and commercial ferments have become the mainstay of fermentation co-culture at this stage. Compound strain fermentation is more focused on a particular product characteristic or equalizing the fermentation characteristics of the fermented product, such as improving or enhancing organoleptic properties, flavor, viscosity, quality, fermentation time, etc. [7]. While regular commercial culture focuses on the overall fermented milk product, special commercial fermenters improve the product characteristics to ensure product quality while enhancing the quality characteristics of the fermented milk, starting from the fundamental nature of the food consumed by the consumer [8]. The high quality of food demanded by consumers and the rapid development of the fermentation industry have led to further exploration and selection of excellent fermentation strains that combine multiple fermentation advantages [9]. *S. thermophilus* has become an indispensable fermenting strain for fermented dairy products due to its certified safety, strong acid production capacity, and fast fermentation speed [10], and most of the compound strains contain *S. thermophilus* in fermented products. The production and evaluation of *S. thermophilus* through genomics, metabolomics, phenotypic analysis, and other research techniques are also being carried out gradually [11]. In this paper, *S. thermophilus* JM905 was isolated from traditional fermentation products; it has a good acid-producing ability, which can be used as a potential industrial fermentation strain, and the study of its fermentation and metabolic characteristics during fermentation has certain practical significance. The selection and exploitation of good fermentation strains are not limited to having certain outstanding characteristics but the overall balance of the final product quality [12]. The screening and mining of excellent fermentation strains are not limited to having certain outstanding characteristics and the overall balance of the final product quality. A better exploration of the fermentation characteristics and metabolite fraction functions of fermented milk at different stages of fermentation and correlation with product quality [13] would be beneficial in facilitating the preparation of fermented milk production and reducing excessive waste of resources.

Therefore, the aim of this study was to further investigate the fermentation characteristics of *S. thermophilus* JM905 with fermentation potential at different stages of fermentation in the milk environment and to analyze the relationship and characteristics between pH, acidity, viscosity, odor, sensory evaluation, and changes in major metabolites of the fermented milk of *S. thermophilus* JM905 by elucidating the utilization of various carbon source substrates. From the perspectives of substrate utilization specificity, fermentation characteristics, and metabolic components in the milk environment, we can provide theoretical data for strains with potential application in the fermentation field and provide a new direction for the selection of excellent fermentation strains.

## 2. Materials and Methods

### 2.1. Strain, Culture Medium, and Growth Conditions

*S. thermophilus* JM905 is isolated from a traditional fermentation product and has good fermentation potential after pre-fermentation experiments. Then, it was inoculated at 5% inoculum in an M17 broth liquid medium (Hopebio biotechnology Co., Ltd., Qingdao, China) (sterilized at 121 °C for 15 min) for activation, and the culture condition was set at a constant temperature of 37 °C for 12 h. The above procedure was repeated 2 times for the generation to ensure the maximum density of viable cells and cultured again in M17 broth liquid medium at the above proliferation rate [14]. Fermentation was carried out with an inoculum of 10^7^ CFU/mL.

### 2.2. Utilization of Carbon Sources by the Strains

The use of carbon sources by the strains was determined by a high-throughput microbial phenotyping system (Biolog Omnilog Technology Co., Ltd., Shanghai, China). After the strain had been incubated for 48 h by scribing, an inoculum suitable for Streptococcus was selected, and a single colony was dipped in an appropriate amount using a disposable sterile cotton swab, which was rubbed close to the top of the inoculum without touching the wall of the inoculum, and the colony tissue was sufficiently ground, inserted into the inoculum, shaken up and down slowly to avoid air bubbles, and adjusted to the appropriate turbidity [15]. After adjusting to the appropriate turbidity, 100 μL of the inoculum with colony tissue was added to each well of the GEN III plate and incubated in the phenotyping system incubator at a temperature of 37 °C for 72 h.

### 2.3. Fermented Milk Sample Preparation

Skim milk powder was added to distilled water at 65 °C, fully homogenized, and sterilized at 95 °C for 20 min [16]. The corresponding mass of powder was weighed and inoculated at 5 × 10^7^ cfu/g into skim milk medium cooled below 40 °C with sucrose, and the fermentation conditions were constant temperature at 37 °C. Monitoring the pH value during fermentation, pH values of F1 (6.0 ± 0.01), F2 (5.1 ± 0.01), and F3 (4.5 ± 0.01) were selected as the indexes for the pre-, mid-, and post-fermentation stages, respectively, based on the data from the pre-fermentation experiments in the previous stage.

### 2.4. Physico-Chemical Analysis

#### 2.4.1. Fermented Milk pH, Acidity, and Post-Acidification

Changes in pH during fermentation were monitored hourly by a fermentation monitor (AMS-ALLIANCE Technology Co., Ltd., Shanghai, China).

A sample of 10 mL per hour is taken and diluted in 20 mL of water to determine the pH. When the pH of the dilution is 8.3, the number of milliliters of NaOH standard solution consumed at 0.1000 moles/litre is calculated and multiplied by 10 to give the acidity of the sample, which is expressed in °T.

#### 2.4.2. Fermented Milk Water-Holding Capacity, Viscosity, and Viable Bacteria Count

A certain amount of fermented sample mass was weighed as *m*_1_, loaded into a centrifuge tube, and centrifuged at 5500 rpm for 30 min at low temperature; then, the supernatant was poured off, inverted for 10 min to allow the supernatant to flow out fully, and finally, the mass of the precipitate was weighed as *m*_2_. The water-holding capacity was calculated as follows:

$$Water\;holding\;capacity(\%)=\frac{m_{2}}{m_{1}}\times 100\%$$

A total of 1 mL of each fermented milk from the pre-fermentation, middle, and post-fermentation periods (F1, F2, and F3) was taken, and serial decimal dilutions were made with 90% saline in a gradient. A total of 1 mL of fermented milk dilution was taken for each sample and incubated in the M17 agar medium incubated in the M17 agar medium of choice at 37 °C for 48 h medium of choice at 37 °C for 48 h. To determine the changes in the number of viable bacteria at different stages in fermented milk [17].

Viscosity was measured in this test by using a DVS+ viscometer with rotor LV-4 (64) to measure the viscosity of the fermented milk at 100 rpm and taking the value at 30 s as a measure of the viscosity of the curd of the strain.

#### 2.4.3. The Odor and Taste of Fermented Milk

Determination was carried out by an electronic nose measuring system (INSENT Technology Co., Ltd., Beijing, China). After cleaning the sensor for 60 s, a disposable sterile needle was inserted into the sample bottle as close as possible to the sample surface but without contact. The sampling interval was 1 s, the determination time was 60 s, and the determination was performed 3 times.

Determination was carried out by an electronic tongue measurement system (INSENT Technology Co., Ltd., Beijing, China). The sensor probe should be soaked in a buffer solution for 24 h and cleaned. The sample is homogenized using a homogenizer, extracted by magnetic stirring for 20 min, mixed thoroughly, and centrifuged at 6500 rpm for 10 min by freezing. Then, the supernatant is extracted using a double layer of medium-speed quantitative filter paper, and the filtrate is diluted to 100 mL and poured into a special beaker for the electronic tongue to be measured 3 times.

### 2.5. Determination of Fermented Milk Protein and Free Amino Acids

The protein content of fermented milk was determined using an automated Kjeldahl nitrogen tester (HITACHI Technology Co., Ltd., Shanghai, China), and all experiments were repeated 3 times in parallel.

After acid digestion, fermented milk was determined using an amino acid analyzer.

### 2.6. Determination of Fermented Milk Fat and Fatty Acids

A quantity of fermented milk sample was taken and diluted using sterile PBS solution, and the fat in it was extracted by hydrolysis ether solution. After hydrolysis, the fat was concentrated to dryness using a rotary evaporator, and the residue was the fat extract, which was subjected to saponification and methyl esterification of fatty acids directly after the addition of the internal standard [18].

The fermented milk was subjected to dilution treatment, and the alkaline (ammonia) hydrolysate of the sample was extracted with anhydrous ether and petroleum ether; the solvent was removed by distillation or evaporation, and the content of the extracted fat dissolved in the solvent was determined [19].

### 2.7. Sensory Evaluation

Sensory evaluation was carried out by 30 sensory assessors in the sensory evaluation room of the Northeast Agricultural University. (15 males and 15 females, 22–27 years of age, none with a clear taste preference). All samples were placed undifferentiated and numbered randomly, and pure water was provided for mouth rinsing between tasting different numbered samples. The following assessment criteria were established: freshness, acidity, sweetness, odor, texture, and acceptability. The sensory assessors were asked to evaluate the taste by using a 9-point scale (9, first sensation on the palate and persistent; 8, accompanied throughout the senses; 7, pleasant taste present; 6, pleasant taste present but not persistent; 5, normal taste and persistent; 4, unpleasant sensation but brief; 3, unpleasant sensation present; 2, unpleasant sensation and persistent; 1, very unpleasant sensation present) assessed after overall sensory scoring.

### 2.8. Metabolic Composition of Fermented Milk

Pipette 100 μL of each of the pre-, mid-, and post-fermentation samples into an EP tube, add 400 μL of extract (methanol: acetonitrile = 1:1 (*V*/*V*), containing isotopically labeled internal standard mixture), vortex and mix for 30 s; sonicate for 10 min (ice water bath); leave at −40 °C for 1 h. Centrifuge samples at 4 °C, 12,000 rpm (centrifugal force 13,800× *g*, radius 8.6 cm). The samples were centrifuged for 15 min; the supernatant was removed from the injection vial and tested on the machine; and all samples were mixed with an equal amount of supernatant to form QC samples for detection by ultra-high performance liquid chromatography (UHPLC). UHPLC (BIOTREE Biotechnology Co., Ltd., Shanghai, China) chromatographic conditions: column temperature 25 °C; flow rate 0.5 mL/min; injection volume 2 μL; mobile phase composition A: water + 25 mM ammonium acetate + 25 mM ammonia; B: acetonitrile; gradient elution program: 0–0.5 min, 95% B; 0.5–7 min, B from Q–TOF mass spectrometry conditions: ESI source setup parameters: sheath gas flow rate of 50 Arb, Aux gas flow rate of 15 Arb, capillary temperature of 350 °C. Arb, capillary temperature 350 °C, full MS resolution 60,000, MS/MS resolution 30,000, collision energy 20/30/40 in NCE mode, injection voltage 3 kV (positive) or −3 kV (negative).

For non-targeted metabolomics studies, the positive and negative ion modes were selected for full analysis, and the positive and negative ion data were integrated for analysis.

### 2.9. Data Analysis

Each experiment was repeated three times independently, and the results are expressed as mean Earth standard deviation (SD). SPSS software (SPSS 18.0, Inc, Chicago, IL, USA) was used to analyze the experimental data. *p*-values < 0.05 were statistically significant. Plots of the experimental data were generated using Origin software (Origin 2018, Inc., Chicago, IL, USA). The non-target metabolism data were analyzed by BIOTREE Biotechnology Co., Ltd., Shanghai, China.

## 3. Results

### 3.1. Utilization of Carbon Sources by Strains

Table S1. Utilization of carbon sources by *Streptococcus thermophilus* JM905 (placed in Supplementary Materials).

**Figure 1:**
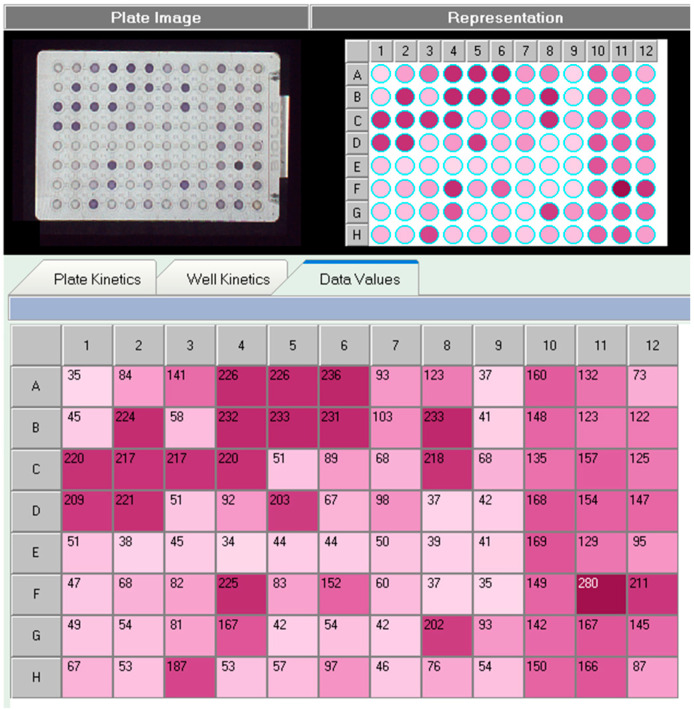
Utilization of substrate carbon sources in the GEN III plate by *S. thermophilus* JM905. The top left image is a real-time image of the Biolog system, and the top right image shows the level of significance of the carbon source utilization, where the numbers in the lower image are numerical displays of the level of significance. The color shades in the picture represent the degree of utilization of the strain to the corresponding carbon source, using the A1 well as a control, each well represents a different kind of carbon source, and the darker the color or the larger the value means the better the utilization effect.

Note: Carbon source utilization test difference is the value of x-A1, i.e., substrate value per well—negative control value; degree of utilization: 0–50 (+), 51–100 (++), 101–150 (+++), 151–200 (++++), 201–250 (+++++). Figure 1. self-generated by the high-throughput microbial phenotyping system.

As shown in Table S1 (placed in Supplementary Materials), as a result, *S. thermophilus* JM905 grew rapidly on D-alginate, D-cellulose, gentian disaccharide, α-D-lactose, β-formyl-glucoside, D-salicin, N-acetyl-D-glucosamine, N-acetyl-D-galactosamine, α-D-glucose, D-mannitol, D-fructose, D-galactose, L-rhamnose, D-sorbitol, D-mannitol, glycerol, D-gluconic acid, and L-malic acid with good utilization, using the A-1 negative control as a reference. Carbon sources are essential for the growth and energy supply of strains, and it is even more important to add carbon sources that can be fully utilized by the strains during the fermentation co-culture process [20].

### 3.2. Changes in pH and Acidity during Fermentation

**Figure 2:**
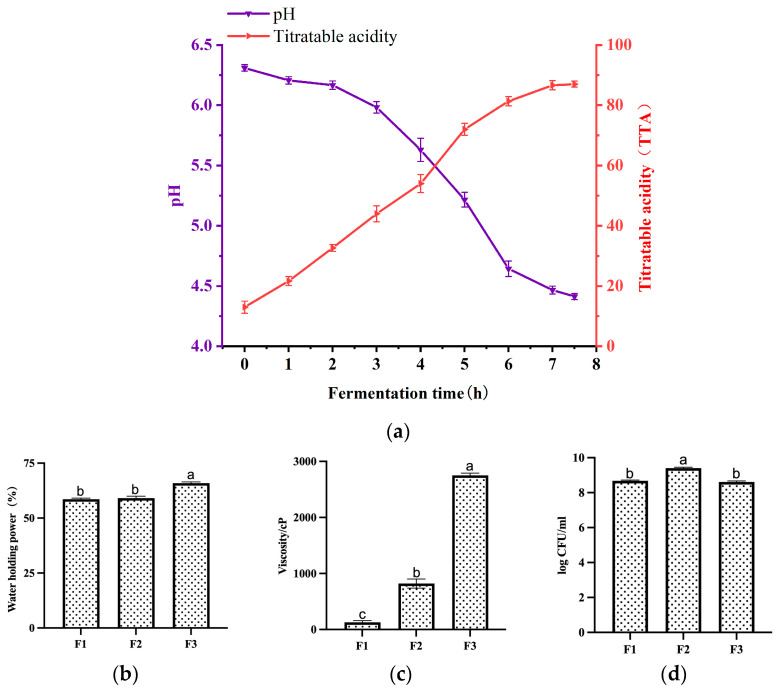
Fermentation characteristics of fermented milk at different periods of fermentation (F1, F2, and F3, respectively). (**a**) Changes in pH and acidity of fermented milk during the fermentation process. (**b**) Changes in water-holding capacity of fermented milk during F1, F2, and F3. (**c**) Changes in fermented milk viscosity during F1, F2, and F3. (**d**) Changes in fermented milk viable bacteria count during F1, F2, and F3. Duncan’s multiple test, *p* < 0.05, indicated by a, b, and c.

The pH and acidity of the fermented milk were measured per hour (Figure 2a). The pH of the fermented milk decreased slowly during the first 3 h. After 3–4 h of fermentation, the pH began to decrease rapidly, with the greatest decrease in pH occurring at 5–6 h. The rate of acid production reached its maximum at approximately 7 h when the pH of the fermented milk was below 4.5. During the fermentation process, the acidity of the fermented milk gradually increased, with the rate of acid production showing the same rate of decrease as the pH at the same fermentation time, with ΔpH = 74 °T at 7.5 h of fermentation.

### 3.3. Changes in Water-Holding Capacity, Viscosity, and Viable Bacteria Count of Fermented Milk at Different Fermentation Stages

Throughout the fermentation period, the water-holding capacity showed a progressive increase, with no significant increase at stage F2 and a more pronounced increase at stage F3 (*p* < 0.05) (Figure 2b). The amount of acid and polysaccharides produced during F1 was low, and the viscosity was basically that of the fermented milk matrix, with a positive correlation between viscosity and fermentation time (*p* < 0.05) (Figure 2c). Throughout the fermentation process, during the period from F1 to F2 to F3, microbial cells showed an increase in the number of viable bacteria first due to the acquisition of various nutrients in the substrate for growth and the use of lactose to produce lactic acid and then decreased again as the acid environment inhibited the growth and reproduction of microbial cells with the accumulation of various acid yields. (Figure 2d). Data are expressed as mean ± standard variance. And the lactic acid content increases during fermentation (Table 1).

### 3.4. Changes in Odor and Taste of Fermented Milk

**Figure 3:**
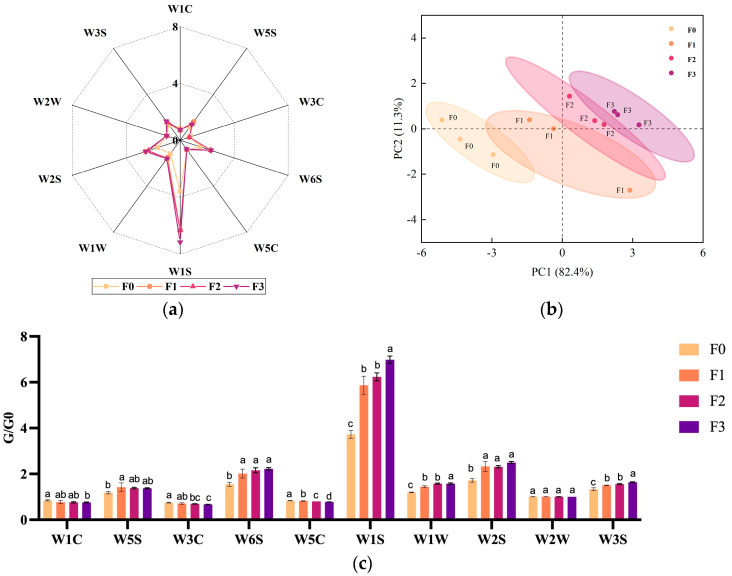

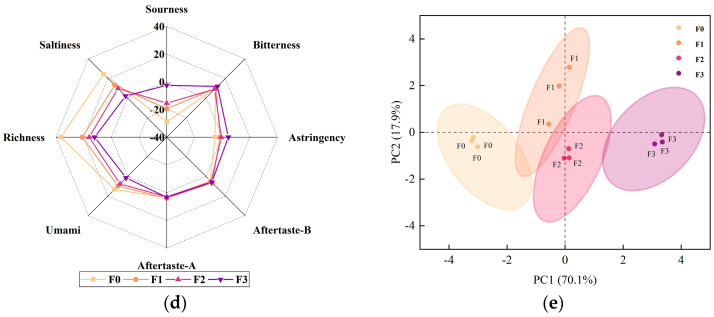
Fermentation characteristics of fermented milk at different periods of fermentation (F0, F1, F2, and F3, respectively; as a comparison, F0 represents the raw odor of unfermented milk). (**a**) Changes in odor values of fermented milk during the F0, F1, F2, and F3 periods. (**b**) Plot of changes in the main components of fermented milk odor in F0, F1, F2, and F3. (**c**) Comparison of the changes in the same odor values detected by the sensors during the periods F0, F1, F2, and F3. (**d**) Variation of fermented milk taste values during the periods F0, F1, F2, and F3; sensors W1C, W5S, W3C, W6S, W5C, W1S, W1W, W2S, W2W, and W3S detected substances as aromatic compounds, nitrogen oxides, ammonia and aromatic compounds, hydrides, olefinic compounds, alkanes, sulfur compounds, alcohol compounds, aromatic compounds (containing sulfur), and aliphatic compounds. (**e**) Plot of the main components of fermented milk taste during F0, F1, F2, and F3. Duncan’s multiple test, *p* < 0.05, indicated by a, b, and c. (The significance in the figure is for each sensor analyzed individually, not a uniform significance analysis for all results.)

The substances In the fermented milk change over time, gradually altering the original taste and smell of unfermented milk and giving it a distinctive taste and smell that belongs to the fermented food (Figure 3a). The sensor detected more significant changes in nitrogen oxides, hydrides, olefinic compounds, alkanes, sulfur compounds, and alcohols and aliphatic compounds in fermented milk at stage F1 compared to F0, with only olefinic compounds decreasing. In stage F3, ammonia, aromatic compounds, and olefins decrease, while alkanes, sulfur compounds, and alcohols continue to increase. Aromatic compounds (containing sulfur) remain largely unchanged throughout the fermentation process (Figure 3c).

The taste richness of unfermented milk was at its highest value, and as fermentation continued, the taste richness, saltiness, and freshness values decreased, and the sourness and astringency values increased, which is in line with the trend of fermentation acid production (Figure 3d). Through electronic nose and tongue detection combined with comprehensive analysis of sensory evaluation, the increase or decrease of a certain type of substance can be used to determine its main nuisance odor source and characteristics, and differences can be analyzed through the determination of the content of components of the same type of substance [21]. This experiment, however, does not show, in detail, the change in the specific compositional content of a substance by annotating only the change in the category of the substance during the fermentation process.

### 3.5. Sensory Evaluation

**Figure 4:**
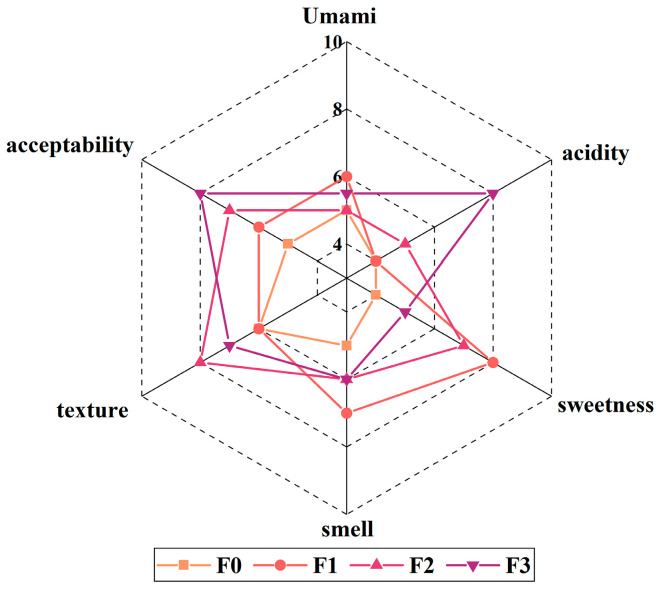
Sensory evaluation of fermented milk at different stages of fermentation.

In contrast to F0, the different stages of fermented milk served to enrich the taste and texture (Figure 4). At stage F1, as acid production is at the beginning and the added carbon source is not yet fully utilized, more lactic and sweet flavors are present, with the odor reaching its highest rating and increasing acceptability, and there is no significant improvement in texture, as the extracellular polysaccharides should be in the process of formation. At stage F2, as acid production continues, the acidity and sweetness are balanced, and the texture is more mellow and homogeneous, reaching its highest value. At stage F3, the gradual increase in acid production rate gives the fermented milk a unique sour taste and, at the same time, changes the texture of the fermented milk from a viscous state to a homogeneous fluid state, but, at the same time, the metabolic utilization of *S. thermophilus* JM905 in the matrix also reduces the milky flavor, with a high level of overall acceptability after a comprehensive evaluation. There was no significant difference in the freshness of the fermented milk throughout the process, analyzing that the freshness of the amino acids due to glycolysis and proteolysis was relatively low and that freshness was not the key taste that distinguished the fermented milk. Overall, the sensory evaluation of the fermented milk was better than F0, indicating that *S. thermophilus* JM905 can enrich the taste and improve the condition of the fermented milk.

### 3.6. Fermented Milk Protein and Free Amino Acid Content

The protein content of fermented milk is 3.78 ± 0.054 g per 100 g, which meets the national regulations for fermented milk protein content. Amino acids are the basic units of protein and are hydrolyzed into various amino acid groups, the content and ratio of which determine the nutritional value of the protein. Also the content of each free amino acid in the fermented milk is shown in Table 2. (-: Indicates that there are no clear criteria for categorisation.) Data are expressed as mean ± standard variance.

### 3.7. Fat and Fatty Acid Content of Fermented Milk

Table 3 shows the fat as well as fatty acid content in fermented milk.

### 3.8. Changes in Key Metabolites of Fermented Milk

**Figure 5:**
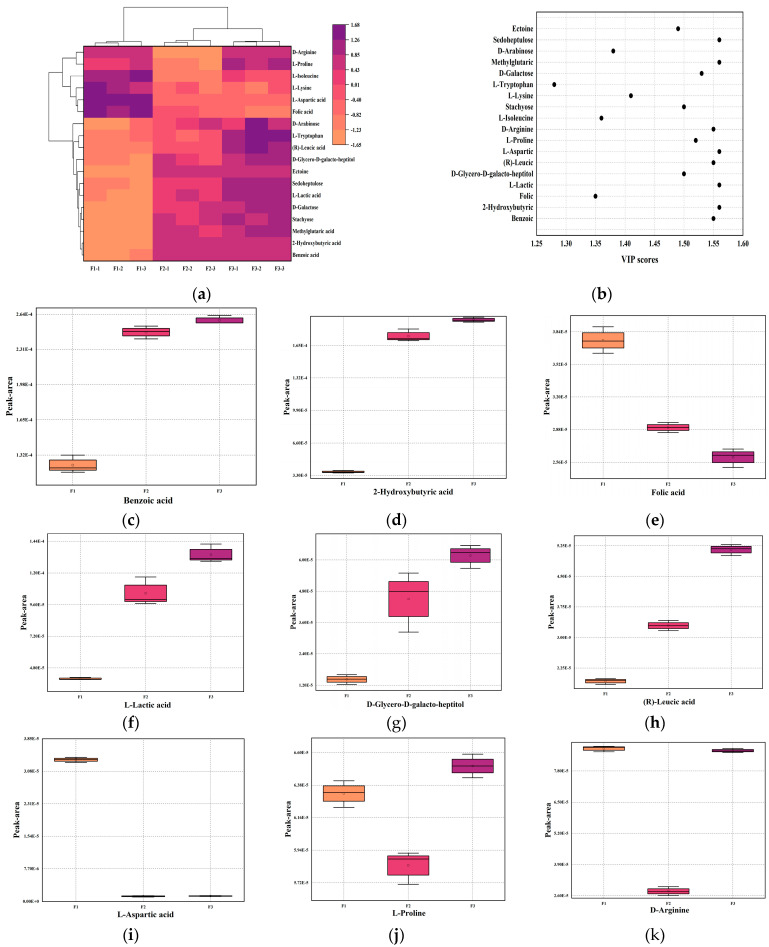

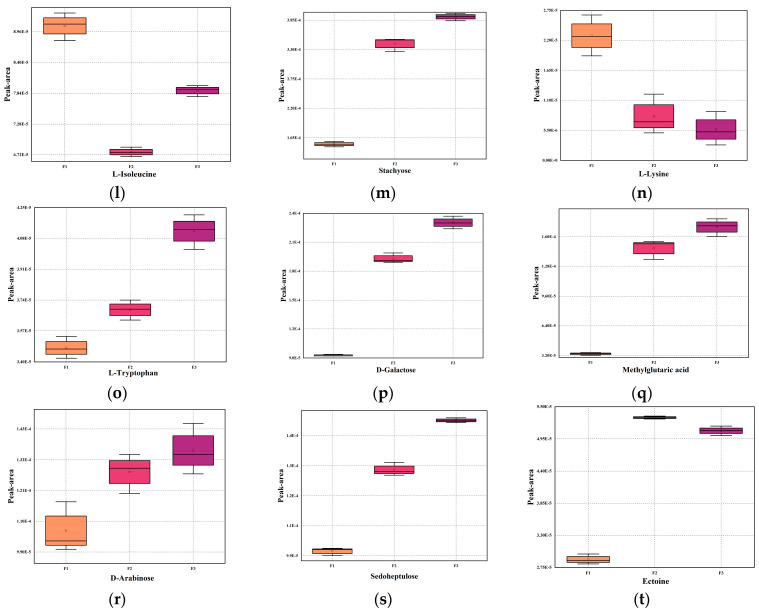
Study of changes in key metabolites in fermented milk at different fermentation periods (F1, F2, F3). (**a**) Heat map and hierarchical clustering of key metabolites in F1, F2, and F3 stages. (**b**) VIP values of each key metabolite throughout the fermentation process. (**c**–**t**) Box-and-whisker diagram of the changes of each key metabolite in stages F1, F2, and F3.

The key metabolites in different stages of fermented milk were benzoic acid, 2-hydroxybutyric acid, folic acid, L-lactic acid, D-glycero-D-galacto-heptitol, (R)-leucic acid, L-aspartic acid, L-proline, D-arginine, L-isoleucine, Stachyose, L-lysine, L-tryptophan, D-galactose, methylglutaric acid, D-Arabinose, Sedoheptulose, and Ectoine. Trends in key metabolite changes are not the same at all stages (Figure 5c–t).

The changes in metabolites annotated to the different stages of fermented milk varied, and inevitably, the characteristic metabolites all varied significantly from one time point to another. This paper is more interested in expressing the trends in metabolically different substances throughout the fermentation process. In this context, the characteristic metabolites were screened and analyzed for their role or physiological function as component substances in the fermentation process.

## 4. Discussion

In recent years, the rise of fermented dairy products has been fueled by a significant increase in consumer attention to food nutrition and safety, prompting a further shift towards higher quality and functionality. It is required not only to expand its application in actual production but also to fully investigate its characteristics during the fermentation process of the corresponding substrates, which needs to be verified by continuous practical operation. Following this, the fermentation characteristics and special metabolites that have been identified are used to target and enhance the nutrients in fermented milk or to conduct other functional studies. The fermentation index is the most intuitive response to the fermentation performance of fermentation strains and the final fermented milk physicochemical state, and the results ultimately determine whether the strain has a strain that can be applied to the actual production. Unsuitable strains or strains with certain fermentation defects may show unsatisfactory results in one of the physicochemical indexes, such as long fermentation time, severe post-acidification of the final fermented milk, or poor curd condition. The key to determining the fermentation rate of a strain is the genetic differences of the strain. Studies have shown that lactate dehydrogenase (LDH) is a key enzyme in the metabolic pathway of lactic acid production by Lactobacillus, which can catalyze the conversion of pyruvic acid into lactic acid. According to the classification of the catalytic products produced, it can be classified into D-lactate dehydrogenase and L-lactate dehydrogenase, which can produce D-lactic acid and L-lactic acid, respectively, whereas the overall tendency of L-lactate is to increase continuously, thus reducing the pH and promoting the fermentation [22]. The fermented milk components and the type of metabolites determine the quality and nutrition of fermented milk. To resolve the deficiency of fermentation strains, it is necessary to supplement the deficiency of single-strain fermentation by compounding more strains, so it is extremely important to explore excellent fermentation strains in the field of fermentation.

Key metabolite components in fermented milk have a significant impact on the quality and fermentation characteristics of fermented dairy products [23]. Non-targeted sequencing technologies allow for a more specific understanding of the composition of individual substances by qualitatively annotating biological information in sample components [24]. Based on the annotated substance composition, specific enhancement or extraction of beneficial components or classes in fermented milk can be performed to improve the quality of different kinds of fermented milk or to conduct corresponding functional studies. Thus, the study of metabolite fractions and trends in fermented milk contributes to a more complete understanding of the quality and fermentation characteristics of a particular strain. The results of the fermentation characterization showed that the pH value of Streptococcus thermophilus JM905 decreased slowly in the early stage of fermentation and changed more significantly in the later stage. A titratable acidity of 87 °T was reached at the completion of fermentation, and *S. thermophilus* JM905 had better acid production characteristics than the same strain fermenting milk [25]. For metabolic annotation to organic acids, benzoic acid, 2-hydroxybutyric acid, folic acid, L-lactic acid, and methylglutaric acid content also increased, which is the same trend as the overall fermentation acid production. The folic acid content continued to decrease, and related studies showed that except for Lactobacillus plantarum, which showed an increase in folic acid content after fermentation, the rest of the strains showed a bottom in folic acid content, which was related to the strain species specificity [26]. In contrast, the results of this paper confirm that the folate content continued to decrease throughout the fermentation process, presumably due to the involvement of other substance production pathways. The key indicator for determining whether fermented milk has reached the fermentation endpoint is pH, which is judged by the amount of acid produced by the strain in the substrate. The results showed a gradual decrease in pH and a gradual increase in acidity with the duration of fermentation, with a more pronounced change in the later stages of fermentation. In an acidic environment with decreasing pH, the degree of protein denaturation gradually increases, and the water retention capacity of *S. thermophilus* JM905 fermented milk increases, which is consistent with the fermentation results and the trend of the above study [27]. The degree of denaturation of proteins in fermented milk, the aggregation of casein in an acidic environment, and the type and content of extracellular polysaccharides are related to the water-holding capacity and viscosity of fermented milk. Increased acid production by *S. thermophilus* JM905 in the fermentation environment leads to protein denaturation in milk. The water-holding capacity of fermented milk is due to the exposure of hydrophobic groups when the proteins in milk are denatured by acid, resulting in reduced diffusivity, intermolecular collisions, and aggregation, leading to an increase in viscosity, and a continuous upward trend in overall viscosity values [28]. Extracellular polysaccharides in fermented milk confer fermented milk viscosity due to the tight junctions and disordered arrangement of monosaccharides. Annotation to the sugar alcohols such as D-glycero-D-galacto-heptitol, D-Arabinose, Sedoheptulose, and others showed a continuous increase in the content of polysaccharides, of which galactose and D-Arabinose were the main constituents constituting the polysaccharides, increasing the degree of viscosity, which is similar to the metabolic annotation results of the present study [29]. The polysaccharide components in improving the texture state in the fermented milk sensory was more obvious in the first and middle stages, which made the milk state appear viscous and pulling, and in the later stage, the texture state appeared to be coagulated and blocky, and it was not possible to discern the effect of its influence.

As for the carbon source utilized by the strain, the results of this paper showed a high utilization of the carbon source D-galactose, which is usually present in the milk matrix as a structural part of lactose, and based on the study of the carbon source of *Streptococcus thermophilus*, it can be hypothesized that D-galactose is under-utilized during fermentation due to the addition of an additional carbon source [30]. The degree of utilization of carbon sources such as sucrose, fructose, mannose, glucose, and galactose by Streptococcus thermophilus was related to the corresponding utilization of the uptake system, as evidenced by the phenotypic traits, which is in agreement with the results of carbon source utilization in this paper [31]. The efficiency of carbon source utilization by Streptococcus thermophilus, as the main strain of fermentation, directly affects its fermentation characteristics and the metabolites presented by its products, regardless of the substrate in which it is fermented and the efficiency or sequence of good utilization of a variety of carbon sources affects the fermentation performance to a certain extent.

The composition of the main substances in fermented milk affects the presentation of flavors. Detecting changes in food odor with biosensors can show the production and trends of different substances and can also be used as a method to detect spoilage of food components (including fats, carbohydrates, and proteins) that cannot be easily removed by the human senses [32]. Organic acids are an important source of odor for fermented lactic acids (e.g., lactic and oxalic acid) [33]. The improvement of taste and odor by *S. thermophilus* JM905 was gradual and continuous. Sourness and alkanes had the most evident correlation with the pH of the fermented milk. The study also showed that as fermented products, the degree of alteration of sourness was primary, followed by freshness [34], which had a positive correlation with glutamic acid and aspartic acid. The intensity of freshness, which is involved in compositional richness, had a lower correlation with the above-mentioned components. It was also found that alkanes, sulfury compounds, and alcohol compounds showed some negative correlation with savory flavor, richness, and freshness. The results of some studies have shown that the production of fatty acids and several specific amino acids are also the main components contributing to changes in acidity. There is a strong correlation between fatty acids and fat content [35]. The results of this experiment showed that various types of fermented milk were low in fat, monounsaturated, and polyunsaturated fatty acids. It has been shown that the fermentation substrate affects the fatty acid content of fermented milk. In this experiment, skimmed milk was chosen as the substrate, which resulted in a lower fat content after fermentation, which in turn affected the fatty acid content. As for the effect of small molecules in fermented milk on sensory evaluation, the addition of free amino acids produced by the fermentation of lactic acid bacteria to low-fat dairy products improves organoleptic palatability after consumption and imparts a creamy flavor and satiety. Different amino acids have different flavors, with aspartic acid and glutamic acid imparting freshness and acidity to fermented milk. Sweet amino acids are less hydrophobic, while bitter amino acids are more hydrophobic, and the corresponding flavor amino acid content affects the flavor of different kinds of fermented milk to different degrees, which together constitute the multi-layered flavor of fermented milk.

Various components are produced by strains during the fermentation process, and the addition of different components can have relevant effects on the fermentation characteristics, metabolites, and potential quality functions of the final fermented milk. This paper focuses more on analyzing the beneficial metabolites, such as polysaccharides and amino acids, that affect the quality and metabolic properties of fermented milk and does not conduct in vitro tests on specific components of fermented milk corresponding to a certain functionality, but functionality tests of fermented milk have also become a hot research topic.

In conclusion, *S. thermophilus* JM905 can enrich milk with a series of nutrients and characteristic metabolites related to the quality and fermentation properties of fermented milk. Research on the metabolites of different kinds of fermented milk and their fermentation characteristics continues, with beneficial metabolites providing additional quality advantages to fermented milk. The addition of beneficial fermentation strains to the original fermented milk also positively affects the content of beneficial metabolites [36]. This lays the foundation for subsequent studies on the corresponding functions and targeted improvement of the quality of fermented products, as well as the quality control of different kinds of fermented milk.

## 5. Conclusions

This study revealed the utilization of various carbon sources by *S. thermophilus* JM905 using a high-throughput microbial analysis system, in which the strain utilized a wide range of carbon sources and made good use of carbon sources such as D-mannitol, D-fructose, D-galactose, L-rhamnose, and D-sorbitol. Throughout the fermentation process, the pH of *Streptococcus thermophilus* JM905 fermented milk showed a consistent opposite acidity increase to the titratable acidity, with a continuous increase in viscosity and water-holding properties, as well as a continuous increase in alkanes, sulfur compounds, and alcohols in terms of odor. There were some differences in fermentation characteristics, organoleptic and metabolite changes at different stages of fermentation, and *S. thermophilus* JM905 fermented milk had a positive effect on the improvement of the nutritional quality and taste of the milk. In addition, the potential physiological functions of functional metabolites in fermented milk under acidic conditions and their interaction effects after consumption still need to be further investigated. The results of the present study provide a reference for further development of excellent fermentation strains to improve the quality of fermented milk and a new direction for screening strains with fermentation potential.

## Figures and Tables

**Table 1:** Changes in lactic acid content of fermented milk with fermentation time.

Fermentation Time (h)	Lactic Acid (g/100 g)
0	1.17 ± 0.18
1	1.95 ± 0.14
2	2.94 ± 0.10
3	3.96 ± 0.24
4	4.86 ± 0.27
5	6.48 ± 0.18
6	7.32 ± 0.14
7	7.8 ± 0.14
7.5	7.83 ± 0.09

**Table 2:** Fermented milk free amino acid content and species.

Free Amino Acid	Type (by Flavor)	Content (g/100 mL)
Aspartic acid	Flavourful amino acid	0.25 ± 0.0036
Glutamic acid		0.91 ± 0.0011
Threonine	Sweetening amino acid	0.14 ± 0.0042
Serine		0.18 ± 0.0040
Glycine		0.062 ± 0.0001
Alanine		0.11 ± 0.0003
Proline		0.32 ± 0.0007
Isoleucine	Bitter amino acid	0.16 ± 0.0023
Leucine		0.31 ± 0.0020
Phenylalanine		0.16 ± 0.0003
Histidine		0.018 ± 0.0002
Tyrosine		0.16 ± 0.0001
Valine		0.19 ± 0.0017
Methionine	-	0.075 ± 0.0002
Lysine		0.25 ± 0.0011
Arginine		0.11 ± 0.0015

**Table 3:** Fat and fatty acid content of fermented milk.

Type	Content (g/100 g)
Fat	0.161 ± 0.0030
Monounsaturated fatty acid	0.0129 ± 0.0022
Polyunsaturated fatty acid	0.0026 ± 0.00015

## Data Availability

Data in the project are still being collected, but all data used in the study are available by contacting the authors.

## Supplementary Materials

The following supporting information can be downloaded at https://www.mdpi.com/article/10.3390/foods12193690/s1. Table S1: Utilization of carbon sources by *Streptococcus thermophilus* JM905.
