# Supplementary material for: Streptococcus thermophilus JM905—Strain Carbon Source Utilization and Its Fermented Milk Metabolic Profile at Different Fermentation Stages
Source: Foods. 2023 Oct 8;12(19):3690. doi: 10.3390/foods12193690 (PMC10572528; doi:10.3390/foods12193690)
Supplement: Supplementary file 1 [file foods-12-03690-s001.zip › foods-2622479-supplementary.pdf]

Table S1

Utilisation of carbon sources by *Streptococcus thermophilus* JM905

Hole	Substrate	Numerical value	Difference (X-A1)	Utilisation+/ inhibition- degree	Hole	Substrate	Numerical value	Difference (X-A1)	Utilisation+/ inhibition- degree
A1	Negative control	35	0	/	E1	Gelatin	51	16	+
A2	Dextrin	84	49	+	E2	Glycl-L-Proline	38	3	+
A3	D-Maltose	141	106	+++	E3	L-Alanine	45	10	+
A4	D-Trehalose	226	191	++++	E4	L-Arginine	34	-1	-
A5	D-Cellulose	226	191	++++	E5	L-Aspartic Acid	44	9	+
A6	Gentiandiose	236	201	+++++	E6	L-Glutamic Acid	44	9	+
A7	Sucrose	93	58	++	E7	L-Histidine	50	15	+
A8	D-Turanose	123	88	++	E8	L-Pyroglutamic Acid	39	4	+
A9	Stachyose	37	2	+	E9	L-Serine	42	7	+
B1	D-Raffinose	45	10	+	F1	Pectin	47	12	+
B2	α-D-Lactose	224	189	++++	F2	D-Galacturonic Acid	68	33	+
B3	Melibiose	58	23	+	F3	L-Galactonic Acid-g-Lactone	82	47	+
B4	β--Methyl-D-Glucoside	232	197	++++	F4	D-Gluconic Acid	225	190	++++
B5	D-Salicin	233	198	++++	F5	D-Glucuronic Acid	83	48	+
B6	N-Acetyl-D-Glucosamine	231	196	++++	F6	Glucuronamide	152	117	+++
B7	N-Acetyl-D-Galactosamine	103	68	++	F7	Mucic Acid	60	25	+
B8	N-Acetyl-β-Galactosamine	233	198	++++	F8	Quinic Acid	37	2	+
B9	N-Acetyl-Neuraminic Acid	41	6	+	F9	Saccharic Acid	35	0	/
C1	α-D-Glucose	220	185	++++	G1	Hydroxyphenyl Acetic Acid	49	14	+
C2	D-Mannose	217	182	++++	G2	Pyruvic Acid methyl Ester	54	19	+
C3	D-Fructose	217	182	++++	G3	D-Lactic Acid Methyl Ester	81	46	+
C4	D-Galactose	220	185	++++	G4	L-Lactic Acid	167	132	+++
C5	3-Methyl glucose	51	16	+	G5	Citric Acid	42	7	+

C6	D-Fucose	89	54	++	G6	a-Ketoglutaric Acid	54	19	+
C7	L-Fucose	68	33	+	G7	D-Malic Acid	42	7	+
C8	L-Rhamnose	218	183	++++	G8	L-Malic Acid	202	167	++++
C9	Inosine	68	33	+	G9	Bromosuccinic Acid	93	58	++
D1	D-Sorbitol	209	174	++++	H1	Tween 40	67	32	+
D2	D-Mannitol	221	186	++++	H2	γ-Amino-Butyric Acid	53	18	+
D3	D-Arabitol	51	16	+	H3	α-Hydroxy-Butyric Acid	187	152	++++
D4	Inositol	92	57	++	H4	β--Hydroxy-D,L-Butyric Acid	53	18	+
D5	Glycerol	203	168	++++	H5	α-Keto-Butyric Acid	57	22	+
D6	D-Glucose-6-Phosphate	67	32	+	H6	Acetoacetic Acid	97	62	++
D7	D-Fructose-6-Phosphate	98	63	++	H7	Propionic Acid	46	11	+
D8	D-Aspartic Acid	37	2	+	H8	Acetic Acid	76	41	+
D9	D-Serine	42	7	+	H9	Formic Acid	54	19	+

Note: Carbon source utilisation test difference is the value of x-A1, i.e. substrate value per well - negative control value; degree of utilisation: 0-50 (+), 51-100 (++), 101-150 (++++), 151-200 (++++), 201-250 (+++++).
